# The Anti-Inflammatory Activity of Curcumin Protects the Genital Mucosal Epithelial Barrier from Disruption and Blocks Replication of HIV-1 and HSV-2

**DOI:** 10.1371/journal.pone.0124903

**Published:** 2015-04-09

**Authors:** Victor H. Ferreira, Aisha Nazli, Sara E. Dizzell, Kristen Mueller, Charu Kaushic

**Affiliations:** 1 Department of Pathology & Molecular Medicine, McMaster University, Hamilton, Ontario, Canada; 2 McMaster Immunology Research Centre, McMaster University, Hamilton, Ontario, Canada; Burnet Institute, AUSTRALIA

## Abstract

Inflammation is a known mechanism that facilitates HIV acquisition and the spread of infection. In this study, we evaluated whether curcumin, a potent and safe anti-inflammatory compound, could be used to abrogate inflammatory processes that facilitate HIV-1 acquisition in the female genital tract (FGT) and contribute to HIV amplification. Primary, human genital epithelial cells (GECs) were pretreated with curcumin and exposed to HIV-1 or HIV glycoprotein 120 (gp120), both of which have been shown to disrupt epithelial tight junction proteins, including ZO-1 and occludin. Pre-treatment with curcumin prevented disruption of the mucosal barrier by maintaining ZO-1 and occludin expression and maintained trans-epithelial electric resistance across the genital epithelium. Curcumin pre-treatment also abrogated the gp120-mediated upregulation of the proinflammatory cytokines tumor necrosis factor-α and interleukin (IL)-6, which mediate barrier disruption, as well as the chemokines IL-8, RANTES and interferon gamma-induced protein-10 (IP-10), which are capable of recruiting HIV target cells to the FGT. GECs treated with curcumin and exposed to the sexually transmitted co-infecting microbes HSV-1, HSV-2 and *Neisseria gonorrhoeae* were unable to elicit innate inflammatory responses that indirectly induced activation of the HIV promoter and curcumin blocked Toll-like receptor (TLR)-mediated induction of HIV replication in chronically infected T-cells. Finally, curcumin treatment resulted in significantly decreased HIV-1 and HSV-2 replication in chronically infected T-cells and primary GECs, respectively. All together, our results suggest that the use of anti-inflammatory compounds such as curcumin may offer a viable alternative for the prevention and/or control of HIV replication in the FGT.

## Introduction

According to the WHO and UNAIDS, women comprise more than half of all people living with HIV-1 [1]. An estimated 40% of all annual global infections occur through HIV invasion of the female genital tract (**FGT**) via exposure to HIV-1 containing semen [2]. The FGT is lined by genital epithelial cells (**GECs**), which are one of the first cells to encounter the virus during sexual transmission. It has been proposed that following exposure, a short phase of local viral amplification in the FGT is necessary for successful establishment of HIV-1 infection [3]. We have previously demonstrated that HIV directly impairs the genital mucosal barrier, leading to viral translocation that could initiate infection of underlying target cells [4, 5]. Thus protecting the mucosal barrier could play a critical role in preventing HIV infection and provide an early window for prophylactic strategies.

The binding of HIV-1 gp120 to GECs results in the upregulation of numerous proinflammatory cytokines, most notably tumor necrosis factor-α (**TNF-α**) through activation of Toll-like receptor (TLR)2 and TLR4 pathways; it is this inflammation that mediates disruption of the mucosal barrier [4, 5]. A number of other studies have demonstrated that inflammation facilitates the acquisition and transmission of HIV-1 infection and contributes to the sequelea of disease associated with chronic infection, including cardiovascular disease, diabetes and neurodegenerative disorders [6]. Studies of latently infected monocyte and T-cell lines have shown that the addition of TNF-α, interleukin-6 (**IL-6**) or IL-1β can activate HIV-1 replication, mediated through the HIV-long terminal repeat (**LTR**) promoter region [7–9]. Furthermore, lower levels of IL-1β, IL-6 and TNF-α were measured in unstimulated peripheral blood mononuclear cells of highly HIV-exposed, persistently HIV-seronegative women, suggesting an *immunoquiescent* phenotype among this resistant cohort [10]. These studies strongly suggest that lower levels of inflammation may decrease susceptibility to HIV-1 and decrease viral replication, perhaps impairing transmission.

A number of studies have shown that sexually transmitted co-infections increase HIV-1 genital shedding and transmission [11]. Herpes simplex virus type 2 (**HSV-2**) is one of the most prevalent viral sexually transmitted infections (STIs), affecting 20–30% of sexually active adults in North America. A recent meta-analysis demonstrated that HSV-2 infection was associated with a threefold increase in susceptibility to HIV in both men and women [12]. In addition to viral co-infections, sexually transmitted bacteria such as *Neisseria gonorrhoeae* have also been suggested to play an important role in enhancing HIV infection or replication [13–18]. GECs are the first cells to come into contact with both HIV and other sexually transmitted pathogens. We previously showed that not only could co-infecting microbes, specifically HSV-1, HSV-2 and *N*. *gonorrhoeae*, directly induce HIV replication in T-cells, but that in response to these pathogens, GECs upregulated inflammatory mediators that *indirectly* enhanced HIV replication [19].

Curcumin (*diferuloylmethane*), the principal curcuminoid of the spice turmeric, is a highly pleiotropic compound that has been shown to possess anti-inflammatory as well as antimicrobial activities [20, 21]. It has been shown to modulate multiple cell signaling molecules such as proinflammatory cytokines (TNF-α, IL-1β, IL-6) and transcription factors such as NFκB and AP-1 [20]. Curcumin has also shown beneficial anti-inflammatory properties in clinical trials for Crohn’s disease and rheumatoid arthritis, among others illnesses, and has been shown to possess anti-HIV activities [22–24].

Given its potent anti-inflammatory properties, we decided to investigate whether curcumin could be used to abrogate inflammatory processes that facilitate HIV-1 acquisition in the FGT or contribute to HIV amplification. Our results suggest that curcumin a) protects the upper genital epithelial barrier against HIV-1-mediated disruption and inflammation, b) prevents the gp120-mediated upregulation of chemokines by GECs that recruit HIV target cells to the FGT, c) blocks co-infection-mediated direct and indirect enhancement of HIV replication in T-cells, d) decreases HIV amplification in chronically infected T-cells directly and e) blocks HSV-2 viral replication in GECs by a mechanism that likely involves NFκB.

## Materials and Methods

### Cell Lines, Viruses and Bacteria

HIV-IIIB was prepared from the chronically infected H9 T-cell line (ATCC, Manassas, VA, USA), followed by virus concentration by Amicon Ultra-15 filtration system (Millipore, Billerica, MA, USA) [4]. All HIV-1 stocks were titred for infectious viral units (**IVUs**)/mL by TZM-bl (ATCC) indicator cell assay, as previously described [25]. Vero cells (ATCC) were maintained in α-MEM media (McMaster University, Hamilton, ON, Canada) supplemented with 5% FBS (Life Technologies Inc., Burlington, ON, Canada), 100 U/mL penicillin/streptomycin (**pen/strep**) (Sigma-Aldrich, Oakville, ON, Canada), 2 μM L-glutamine (**L-glu**) (Life Technologies Inc.), and 10 μM of 4-(2-hydroxyethyl)-1-piperazineethanesulfonic acid (**HEPES**) (McMaster University). HSV-1 KOS [26] and HSV-2 333 [27] viral stocks were prepared by infecting Vero cells at a multiplicity of infection (MOI) of 0.01 for 24–48 hours. HSV-1 KOS was a kind gift from Dr. Karen Mossman (McMaster University). *N*. *gonorrhoeae* clinical strain 2071, a kind gift from Dr. Scott Gray-Owen (University of Toronto), was grown at 37°C in a 5% CO_2_ humidified incubator from frozen stocks on GC agar base supplemented with 1% IsoVitaleX enrichment (BD, Mississauga, ON, Canada). 1G5 Jurkat T cells [28], a kind gift from Dr. Gray-Owen, were maintained in RPMI (McMaster University) media supplemented with 10% FBS, 100 U/mL pen/strep, 2 μM L-glu and 10 μM of HEPES [19].

### Source of Tissues and Epithelial Cell Preparation

Endometrial and endocervical tissues were obtained, following written informed consent, from women undergoing hysterectomies for non-malignant gynecological purposes at McMaster University Medical Centre in Hamilton, ON, Canada. This study was approved by the Hamilton Health Sciences-McMaster University Research Ethics Board. The protocol for isolation, culture and assessment of primary GEC culture purity are described elsewhere [29, 30]. Briefly, endometrial and endocervical tissues were minced into small pieces and digested in an enzyme mixture and GECs were isolated by a series of separations through nylon mesh filters (Small Parts, Inc. Logansport, IN, USA). Approximately 1x10^5^ GECs were seeded onto transwell inserts (BD) and grown until they formed confluent monolayers, as measured by a trans-epithelial resistance (**TER**) >1 kΩ/cm. The purity of GEC monolayers was between 95 and 98%, with no trace of any hematopoietic cells [29]. TERs were measured before exposing the cells to HIV or gp120 (pre-treatment TER) and 24 hours after, and expressed as a percent of pre-treatment TER [4].

### Confocal Microscopy

Confluent primary endometrial and endocervical GECs were grown on transwell culture inserts and pre-treated with 5 or 50 μM curcumin (Sigma-Aldrich) or primary cell culture media [31] as control for 1 hour prior to exposure to 10^5^ IVUs of HIV, recombinant gp120 (Immunodiagnostics, Woburn, MA, USA) at 0.1μg/mL or primary cell culture media (mock infection). This range of concentration of curcumin was selected because it was sufficient to block the induction of TNF-α from lipopolysaccharide (LPS)-stimulated monocytes [32]. The recombinant HIV-1 gp120 protein was endotoxin free and viral stock preparations were determined to be free of TNF-α, IL-6, IL-8, and other cytokines via the multiplex bead-based sandwich immunoassay (Luminex Corporation, Austin, TX), described elsewhere [4, 5]. At 24 hours post exposure, GECs were fixed, permeabilized and stained as described before [4]. Imaging was done on an inverted confocal laser-scanning microscope (LSM 510, Carl Zeiss Canada Ltd., Toronto, ON, Canada). For each experiment, confocal microscope settings for image acquisition and processing were identical between controls and treated monolayers and 3 random images were acquired and analyzed for each experimental condition.

### Cytokine and Chemokine Measurement

Supernatants were collected and TNF-α, IL-6, interferon-gamma-inducible protein-10 (**IP-10**), eotaxin, monocyte chemotactic protein-1 (**MCP-1**), macrophage inflammatory protein-1α (**MIP-1α**), IL-8 and regulated on activation, normal T-cell expressed and secreted (**RANTES**) were measured using the Magpix multi-analyte technology system (Millipore, Billerica, MA, USA), as per the manufacturer’s instructions. In some experiments, TNF-α was measured in GEC cell culture supernatants by ELISA (R&D Systems, Minneapolis, MS, USA).

### Measuring HIV Replication in Chronically Infected T-cells

5x10^5^ chronically HIV-infected H9 T-cells were exposed once or daily to 5 or 50 μM curcumin or serum-free RPMI as control At several time points post-treatment, supernatants were collected and HIV-1 p24-antigen was measured using a commercial p24 ELISA kit (Zeptometrix Corp., Buffalo, NY, USA), as per the manufacturer’s instructions. Alternatively, 1x10^5^ chronically infected H9 T-cells were treated with 5 or 50 μM curcumin for 1 hour and subsequently exposed to TLR ligands for 24 hours. TLR ligands included the TLR3 ligand poly I:C (25 μg/mL; Sigma-Aldrich), the TLR4 ligand LPS from *Escherichia coli* (100 μg/mL Sigma-Aldrich) and the TLR5 ligand flagellin from *Salmonella typhimurium* (10 μg/mL; Alpha Diagnostic, San Antonio, TX, USA). TLR ligand concentrations were selected according to previous studies, described elsewhere [5, 19, 33].

### Indirect Activation of the HIV-LTR in 1G5 Cells

1G5 Jurkat T-cells were used to measure trans-activation of the HIV-LTR promoter by epithelial cell innate inflammatory responses [15, 19]. Briefly, confluent primary endometrial GECs were pre-treated with or without curcumin for 1 hour and immediately exposed to 10^4^ PFUs of HSV-1 KOS or HSV-2 333 or 10^6^ colony-forming units (CFUs) of *N*. *gonorrhoeae* strain 2071 for 2 hours. After removing the inoculum, cells were washed 5 times with PBS; fresh media was added to the cells and supernatants were collected 24 hours post-exposure. To ensure that there was no residual live HSV, supernatants were exposed to 1 cycle of UV energy (10kJ) (Agilent, Santa Clara, CA, USA). Supernatants collected from *N*. *gonorrhoeae*–infected GECs were filter-sterilized using a 0.2-μm filter (BD) to remove any residual bacteria. The supernatants were then incubated with 10^6^ 1G5 cells for 24 hours, and luciferase activity in the T-cells was measured using a luciferase assay (Agilent) in accordance with the kit’s instructions.

### Measuring HSV-2 Shed Virus in Cell Culture Supernatants

Primary endometrial GECs were grown to confluency and were pre-treated with primary cell culture media (control) or 5 or 50 uM of curcumin, the an NFκB inhibitor pyrrolidine dithiocarbamate (**PDTC**) (Sigma-Aldrich), the p38 MAP kinase inhibitor SB203580 (Invivogen, San Diego, CA, USA) or SP600125 (Invivogen), an inhibitor of c-Jun N-terminal kinase (JNK) for 1 hour, prior to exposure to 10^4^ PFU of HSV-2 strain 333 for 2 hours. Virus-exposed cells were washed with PBS and fresh media was added to the cells. At 24-hours post-infection, apical cell culture supernatants were collected and the amount of shed HSV-2 was measured using a standard Vero plaque assay, as previously described [34].

### Cell Viability Assay

Cell viability was assessed by trypan blue exclusion assay [35]. Primary GECs exposed to curcumin, PDTC, SB203580 or SP600125 were treated with 1x trypsin for 5 minutes at 37°C to detach the cells from the transwell inserts and were pelleted down, and resuspended in primary cell media. Chronically infected H9 T-cells treated with curcumin and 1G5 T-cells exposed to supernatants from primary endometrial GECs exposed to HSV-1, HSV-2 or *N*. *gonorrhoeae* were pelleted down and resuspended in 10% RPMI media. Resuspended primary GECs, H9 cells and 1G5 cells were exposed to trypan blue dye (Life Technologies Inc.) in a 1:1 ratio of cell suspension to dye. Cells were loaded onto a hemocytometer and counted using a light microscope. Cell viability, in terms of percent of live cells, was assessed by subtracting the percent of blue (dead) cells from the total number of cells.

### Statistical Analysis

GraphPad Prism version 5 was used to compare three or more means using a two-tailed Mann-Whitney test or the Kruskal-Wallis non-parametric analysis of variance test, where appropriate. With respect to the Kruskal-Wallis test, when an overall statistically significant difference was measured (p<0.05), Dunn’s test was used to correct for multiple comparisons.

## Results

### Curcumin blocks the disruption of endometrial epithelial cell tight junction proteins and protects the epithelial barrier against HIV-1 gp120

**Fig 1:**
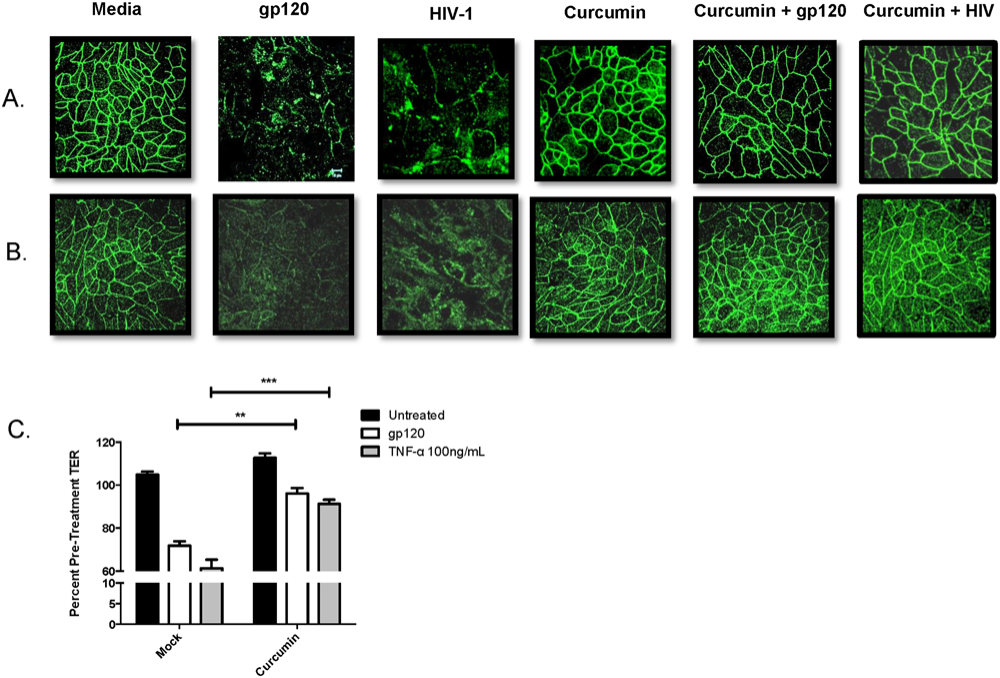
Curcumin prevents tight junction disruption and breakdown of endometrial epithelial barrier integrity caused by HIV-1 gp120. Primary endometrial GECs were grown to confluency and pre-treated with 5μM curcumin or media as control for 1 hour and exposed to 10^5^ IVU of HIV-IIIB, recombinant gp120 at 0.1μg/mL or media (mock infection). At 24 hours post exposure, GECs were fixed and stained for ZO-1 (A) or occludin (B). Imaging was done on an inverted confocal laser-scanning microscope. Alternatively, GECs were exposed to media control (untreated), gp120 at 0.1μg/mL or recombinant TNF-α at 100 ng/mL (positive control) for 24 hours, after which TERs were measured and the percent of pre-treatment TER was calculated (C). Data shown represents the mean ± SEM of three separate experiments. A minimum of two replicates per experimental condition was included in every experiment. Data was analyzed by Kruskal-Wallis non-parametric analysis of variance with Dunn’s test to correct for multiple comparisons. **p<0.01, ***p<0.001. gp120: glycoprotein 120; HIV-1: human immunodeficiency virus-1; TER: trans-epithelial electrical resistance; TNF-α: tumor necrosis factor-α.

Previously we have shown that in the presence of HIV gp120, tight junction (TJ) proteins, including ZO-1 and occludin, expressed by primary GECs, are downregulated due to innate inflammation, leading to the disruption of barrier integrity and subsequent microbial translocation [4, 5]. Given the potent anti-inflammatory effects of curcumin, we decided to measure whether curcumin could block the barrier disrupting effects of HIV gp120. In the presence of HIV-1 or recombinant gp120, ZO-1 (Fig 1A) and occludin (Fig 1B) protein expression in endometrial GEC monolayers was disrupted, which was not seen in monolayers pre-treated with curcumin. Curcumin on its own had no visible effect on TJ protein expression. Pre-treatment with curcumin also prevented HIV gp120- and TNF-α-mediated decrease in TER (Fig 1C), a measure of epithelial barrier integrity [4]. These results suggest that curcumin can prevent HIV-mediated mucosal barrier impairment by maintaining TJ protein expression and maintaining TER in endometrial GECs.

### Curcumin prevents the induction of proinflammatory cytokines associated with barrier disruption

**Fig 2:**
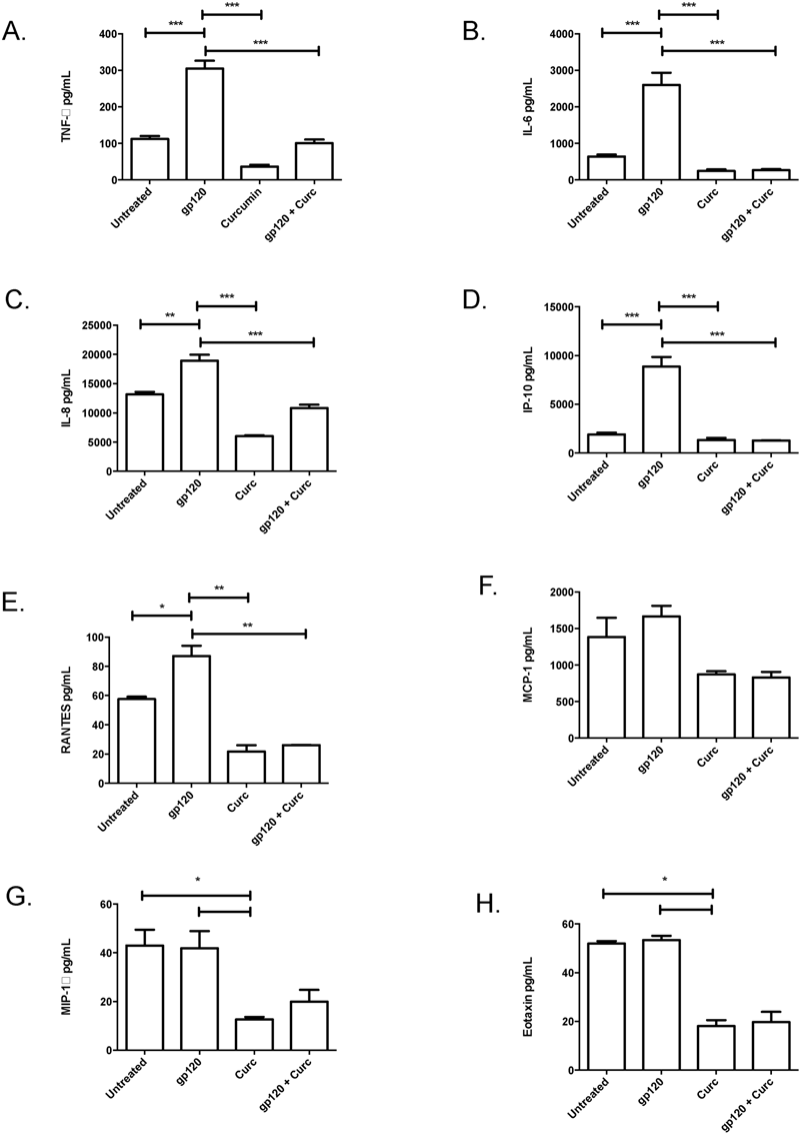
Curcumin prevents the gp120-mediated induction of proinflammatory cytokines or chemokines that recruit HIV-target cells. Confluent primary endometrial GECs were pre-treated with 5μM curcumin or media (untreated) for 1 hour after which the cells were exposed to recombinant gp120 at 0.1 μg/mL. Apical cell culture supernatants were collected at 24 hours post-exposure and proinflammatory cytokines TNF-α (A) or IL-6 (B), as well as the chemokines IL-8 (C), IP-10 (D), RANTES (E), MCP-1 (F), MIP-1α (G) and eotaxin (H) were measured by Magpix multi-analyte assay. Data shown represents the mean ± SEM of three separate experiments. A minimum of two replicates per experimental condition was included in every experiment. Data was analyzed by Kruskal-Wallis non-parametric analysis of variance with Dunn’s test to correct for multiple comparisons *p<0.05, **0.01, ***p<0.001. gp120: glycoprotein 120; HIV-1: human immunodeficiency virus-1; TNF-α: tumour necrosis factor-α; IL: interleukin; IP-10: interferon gamma-inducible protein-10; RANTES: regulated on activation, normal T-cell expressed and secreted; MCP-1: monocyte chemotactic protein-1; MIP-1α; macrophage inflammatory protein-1α; Curc: curcumin.

Previously we have shown that epithelial cell exposure to HIV gp120 results in the upregulation of inflammatory mediators, including TNF-α [4, 5], which mediate the disruption of TJ proteins. Therefore, we sought to determine whether the gp120-mediated upregulation of proinflammatory mediators could be blocked by curcumin. At 24-hours following exposure to recombinant gp120, TNF-α (Fig 2A) and IL-6 (Fig 2B) were significantly upregulated by endometrial GECs relative to untreated GEC controls. Pre-treatment with curcumin abrogated gp120-mediated induction of proinflammatory cytokines, suggesting that curcumin may block the inflammation associated with HIV-mediated mucosal barrier disruption.

**Fig 3:**
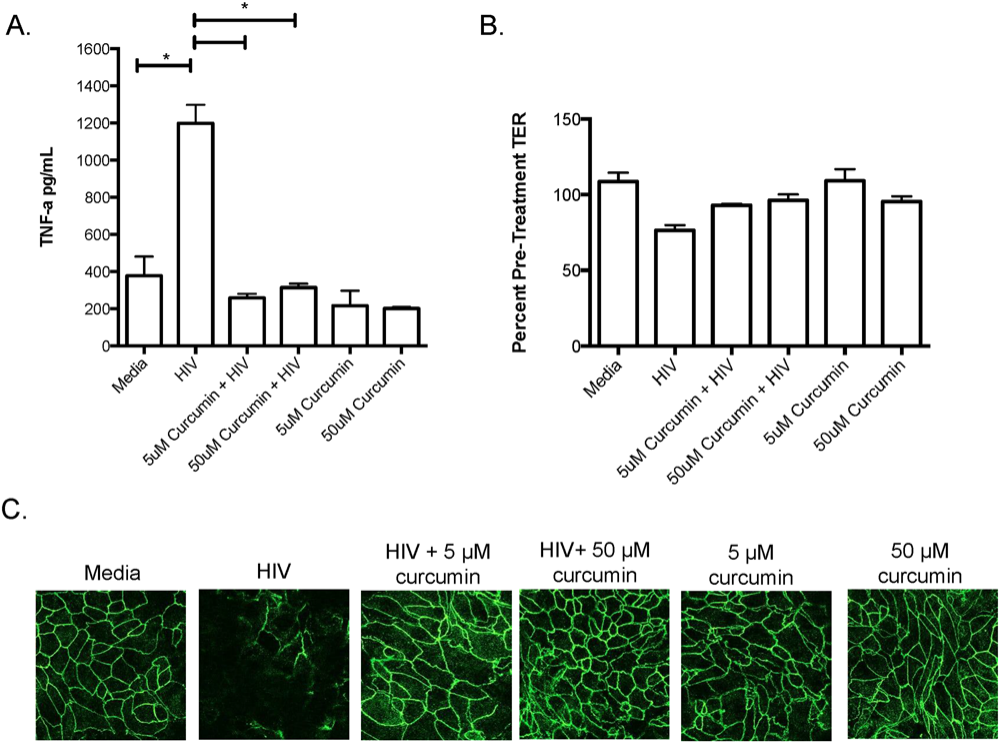
Curcumin protects the endocervix against the barrier-breaking effects of HIV-1. Primary endocervical GECs were grown to confluency and pre-treated with curcumin or primary cell media as control. Monolayers of GECs were exposed to 10^6^ IVU of HIV-IIIB or media (as mock infection). At 24 hours post exposure, cell-culture supernatants were collected and TNF-α was measured by ELISA (A). TERs were measured and the percent of pre-treatment TER was calculated (B) and endocervical monolayers were fixed, stained for ZO-1 and analyzed by immunofluorescent microscopy (C). Imaging was done on an inverted confocal laser-scanning microscope. Data shown represents the mean ± SEM of three separate experiments. Data was analyzed using a two-tailed Mann-Whitney test for non-parametric data. *p<0.05. A minimum of two replicates per experimental condition was included in every experiment performed. HIV: human immunodeficiency virus; TNF-α: tumour necrosis factor-α.

To determine whether curcumin protected the endocervix in a similar manner to endometrium, since this site has been directly implicated in simian immunodeficiency virus (SIV) acquisition in non-human primate models and HIV infection in humans [36, 37], we treated primary human endocervical monolayers with curcumin and exposed them to HIV-1 for 24-hours and measured TNF-α production, TER and ZO-1 expression. Similar to endometrial GECs, endocervical GECs exposed to HIV-1 secreted significantly elevated TNF-α, which was inhibited by curcumin pre-treatment (Fig 3A). Furthermore, endocervical monolayer integrity, as indicated by TER and ZO-1 TJ staining, decreased following exposure to HIV-1 but remained intact among cells exposed to curcumin (Fig 3B and 3C). These results suggest that both endometrial and endocervical epithelial cells can be protected against the barrier-damaging effects of HIV-1 gp120 by curcumin.

### Curcumin blocks the gp120-mediated induction of chemokines associated with the recruitment of HIV-target cells

HIV target cells, which include CD4^+^ T-cells, macrophage and dendritic cells (**DCs**), are recruited to the FGT via chemokine gradients that include MCP-1, MIP-1α, IP-10, eotaxin, IL-8 or RANTES produced by resident tissue cells, such as GECs [4, 5]. Exposure of endometrial GECs to gp120 resulted in significant upregulation of IL-8, IP-10 and RANTES (Fig 2C–2E) but not MCP-1, eotaxin or MIP-1α (Fig 2F–2H). Interestingly, gp120-mediated chemokine induction was abrogated in endometrial GEC monolayers pretreated with curcumin. These results show that pre-treatment with curcumin blocks gp120 mediated upregulation of chemokines that can recruit HIV target cells to the FGT.

### Curcumin pre-treatment blocks HIV amplification in chronically infected T-cells

**Fig 4:**
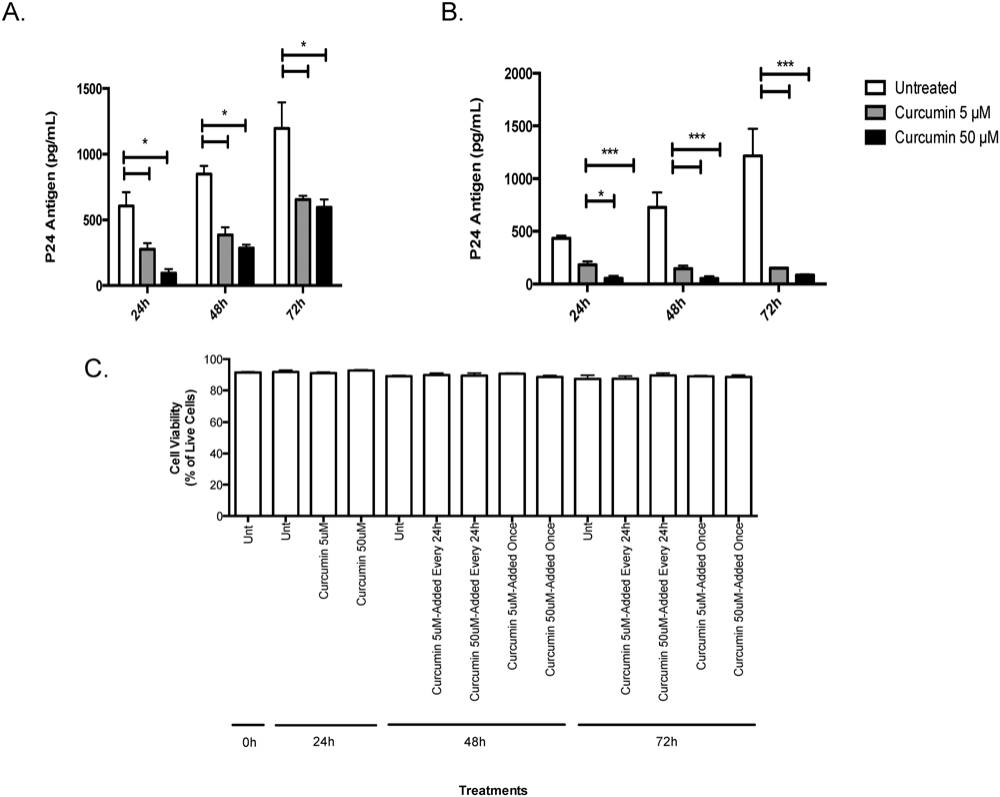
Curcumin prevents the amplification of HIV-1 in chronically infected T-cells. 5x10^5^ chronically HIV-infected H9 T-cells were exposed once to 5 or 50 μM curcumin or serum free RPMI as control. At 24, 48 or 72 hours post-treatment, supernatants were collected and HIV-1 p24-antigen was measured using a commercial p24 ELISA kit (A). Alternatively, 5x10^5^ H9 T-cells were exposed to curcumin once every 24 hours at 5 or 50 μM curcumin after which supernatants were collected and HIV-1 p24-antigen was measured (B). Chronically infected H9 cells were treated with or without curcumin (5 and 50 μM) once every 24 hours, or just once and monitored from 24–72 hours post-treatment. Dead cells were counted following trypan blue exclusion assay using a hemocytometer (C). Data is shown as the percent of live cells in culture. Data shown represents the mean ± SEM of three separate experiments. A minimum of four replicates per experimental condition was included in every experiment performed. Data was analyzed by Kruskal-Wallis non-parametric analysis of variance with Dunn’s test to correct for multiple comparisons *p<0.05, ***p<0.001. Unt: untreated.

Next, we determined if curcumin could directly block HIV replication in chronically infected T-cells. Chronically infected H9 T-cells exposed to a single curcumin treatment secreted significantly less virus into cell culture supernatants relative to untreated cells. After 24 hours of exposure, the effect of curcumin started to diminish but HIV replication remained significantly lower than untreated cells (Fig 4A). Interestingly, when H9 T-cells were exposed to curcumin once daily, HIV levels remained significantly suppressed relative to controls (Fig 4B). Both low (5 μM) and high (50 μM) curcumin doses appeared to be effective in controlling HIV replication, indicating that curcumin could have a potent effect on HIV replication within infected T cells. Chronically infected T-cells remained viable (>90%) throughout the experiment and curcumin-treatment, at either at 5 or 50 μM concentration, or applied once or every 24-hours, did not effect the viability of the cells (Fig 4C).

### Curcumin pre-treatment blocks co-infection mediated induction of the HIV promoter and HIV replication in T-cells

**Fig 5:**
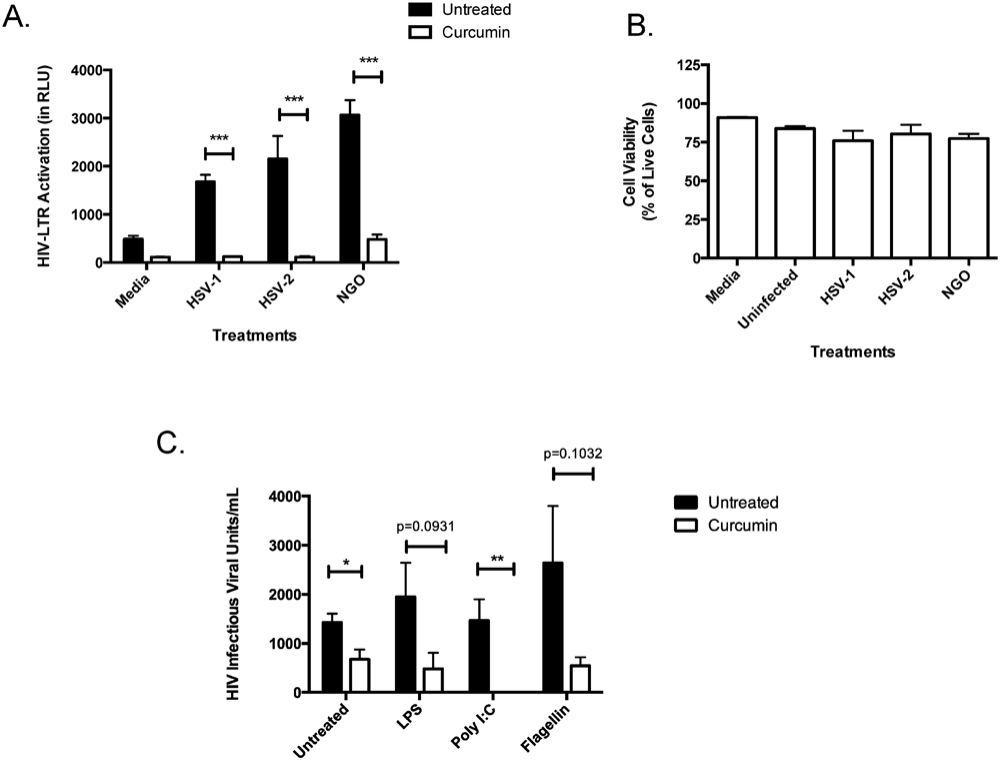
Curcumin can prevent co-infection mediated activation of the HIV-LTR promoter and induction of HIV replication in T-cells. Confluent primary endometrial GECs were pre-treated with 5 μM curcumin or primary cell media (untreated) for 1 hour after which the cells were exposed to 10^4^ PFU of HSV-1, HSV-2 or 10^6^ CFU of *N*. *gonorrhoeae* for two hours. The inoculum was removed and the cells were washed with PBS and subsequently replenished with fresh media. Supernatants were collected 24-hours post-exposure, inactivated to kill live virus or filter sterilized to remove bacterial cells, and subsequently incubated with 10^6^ 1G5 cells for 24 hours, after which the cells were lysed and luciferase activity was measured as a readout for HIV-LTR activation (A). To assess 1G5 cell viability following exposure to conditioned GEC media, 1G5 cells were exposed for 24 hours to RPMI control (Media), primary cell media from uninfected GECs (Uninfected) and conditioned primary cell media from GECs exposed to HSV-1, HSV-2 or *N*. *gonorrhoeae* for 24 hours. Following incubation, cells were collected and cell viability was calculated using the trypan exclusion assay. Results are reported as the percent of live cells in culture (B). 1x10^5^ chronically infected H9 T-cells were pre-treated with 50 μM of curcumin for 1 hour and subsequently exposed to poly I:C, LPS or flagellin for 24 hours. Supernatants were collected and IVUs were measured using the TZM-b1 assay (C). Data shown represents the mean ± SEM of three separate experiments. A minimum of two replicates per experimental condition were included in every experiment performed. Data was analyzed using a two-tailed Mann-Whitney test for non-parametric data. *p<0.05, **p<0.01, ***p<0.001. HIV-LTR: human immunodeficiency virus long-terminal repeats; RLU: relative light units; HSV: herpes simplex virus; NGO: *Neisseria gonorrhoeae*.; LPS: lipopolysaccharide.

Previous studies from our group have shown that in the presence of co-infecting STIs, specifically HSV-1, HSV-2 or *N*. *gonorrhoeae*, primary GECs secrete inflammatory factors that cause *indirect* activation of the HIV-LTR promoter in T-cells, a process synonymous with HIV replication [19]. Therefore, we sought to determine whether blocking inflammatory pathways in primary GECs using curcumin could abrogate indirect induction in T-cells. Cell culture supernatants collected from GECs pretreated with curcumin and exposed to co-infecting STIs were unable to indirectly activate the HIV-LTR promoter in 1G5 T-cells (Fig 5A). In contrast, supernatants collected from untreated GECs exposed to co-infecting microbes potently induced HIV-LTR activation in 1G5 T-cells, suggesting that pre-treatment with curcumin blocks co-infection-mediated inflammation that may increase HIV replication. Exposing 1G5 T cells to conditioned GEC media did not affect cell viability (Fig 5B).

In previous studies, we also demonstrated that TLR ligands, which are representative of various viral and bacterial microbes, could directly activate the HIV-LTR promoter in 1G5 T-cells [19]. To determine whether curcumin treatment was capable of inhibiting the production of infectious virus within infected T-cells, chronically infected H9 T-cells were exposed to TLR ligands in the presence or absence of curcumin. Supernatants were collected and HIV replication was assessed via TZM-b1 assay, which detects infectious live virus [4]. Poly I:C (TLR3 ligand), LPS (TLR4 ligand) and flagellin (TLR5 ligand) all increased infectious HIV production from H9 T-cells, relative to unstimulated controls (Fig 5C). Interestingly, curcumin exposure abrogated TLR-mediated direct induction of HIV replication in T-cells.

Together our results suggest that intact co-infecting microbes, as well as conserved microbial ligands, are capable of inducing HIV promoter activation and production of infectious HIV virions in T-cells and that curcumin pre-treatment can abrogate such potentiation of HIV replication.

### Curcumin blocks HSV-2 replication

**Fig 6:**
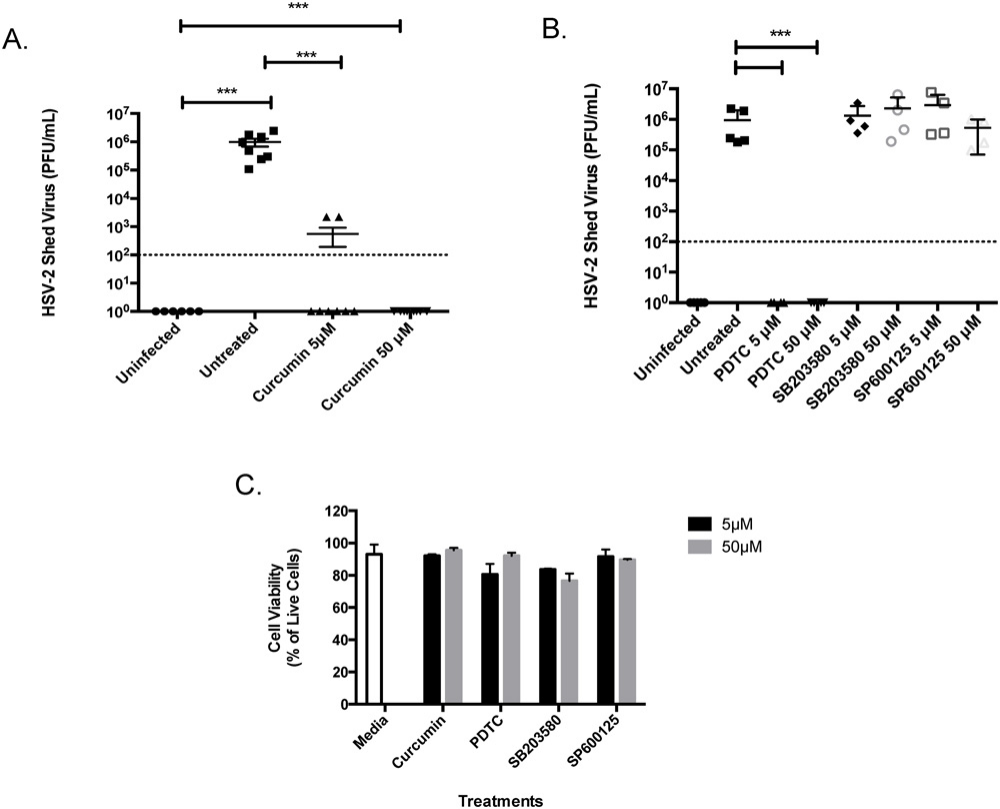
Inhibition of inflammatory signalling pathways decreases HSV-2 viral shedding. Primary GECs were grown to confluency and were pre-treated with media (untreated) or 5 or 50 μM of curcumin (A) or PDTC the NFκB inhibitor, SB203580 a p38 MAP kinsase inhibitor or SP600125 an inhibitor of c-Jun N-terminal kinase (JNK) (B) for 1 hour. The cells were then exposed to 10^4^ PFU of HSV-2 strain 333 for 2 hours. At 24-hours post-infection, apical cell culture supernatants were collected and the amount of shed HSV-2 was measured using a standard Vero plaque assay. To assess whether the inhibitors used in this study affected cell viability (C), primary GECs were exposed to media control, or curcumin, PDTC, SB203580 or SP600125 at 5 or 50 μM for 1 hour. Following exposure, cells were collected and cell viability was calculated using the trypan exclusion assay. Results are reported as the percent of live cells in culture. Data shown represents the mean ± SEM of three separate experiments. A minimum of two replicates per experimental condition was included in every experiment performed. Data analyzed using the Kruskal-Wallis non-parametric analysis of variance with Dunn’s test to correct for multiple comparisons. ***p<0.001. HSV-2: herpes simplex virus type 2; PFU: plaque-forming units; PDTC: pyrrolidine dithiocarbamate.

HSV-2 infection is associated with a threefold increase in susceptibility to HIV in both men and women [38–40] and controlling HSV-2 replication has been associated with decreased HIV replication [41]. Thus, we decided to test the anti-herpetic activity of curcumin in primary human GECs. In cells pretreated with 5μM of curcumin, HSV-2 shed approximately 1,000-fold less virus relative to untreated primary cell controls infected with HSV-2 (Fig 6A). At 50μM of curcumin, no HSV-2 virus could be detected in cell culture supernatants. Since curcumin blocks various inflammatory pathways [20], we pre-treated GECs with inhibitors of curcumin-regulated transcription factors including NFκB (PDTC), p38 MAP kinase (SB203580) or c-Jun N-terminal kinase (JNK) (SP600125), prior to infecting the cells with HSV-2. Our results show that the NFκB inhibitor PDTC completely blocked viral shedding in primary GECs exposed to HSV-2 (Fig 6B), whereas blocking p38 MAP kinase or JNK pathways did not have any effect on HSV-2 viral replication. We also tested the toxicity of the inhibitors on primary GECs and found no significant differences with respect to cell viability between the cells treated with inhibitors compared to media alone treated cells or between treatments with different inhibitors (Fig 6C).

## Discussion

We have described in this study the multiple pathways by which curcumin may have a beneficial role in protecting the FGT against HIV-1. Our results show that curcumin has pleiotropic effects and its protection of barrier function is primarily mediated by its potent anti-inflammatory activities. Curcumin prevented endometrial and endocervical mucosal barrier impairment by HIV-1 gp120 and abrogated gp120-mediated upregulation of proinflammatory cytokines and chemokines. Furthermore, curcumin pre-treatment of primary GECs prevented co-infection mediated upregulation of HIV-LTR activation and replication in T-cells. Finally, we found that curcumin decreased HIV-1 and HSV-2 replication in T-cells and GECs, respectively.

In the present study, we examined primarily the anti-inflammatory activity of curcumin and how this could affect HIV interactions with the genital epithelium and T-cells in the genital tract. Previously, curcumin has been shown to exert potent anti-inflammatory activities such as suppressing the production of proinflammatory cytokines, including TNF-α, IL-1β and IL-6 [20, 42]. Curcumin (at a concentration of 5 uM) has been shown to inhibit LPS-induced production of TNF-α by the human monocytic macrophage cell line, Mono Mac 6, and reduce the biological activity of TNF-α in a fibroblast cell assay [32]. *In vitro*, curcumin modulates the inflammatory response by directly binding to and downregulating the activity of cyclooxygenase-2, lipoxygenase, inducible nitric oxide synthase, NFκB and AP-1, among others [43]. In the current study, we observed that curcumin pre-treatment significantly decreased gp120-mediated induction of proinflammatory cytokines and chemokines and that curcumin protected the epithelial barrier against the disrupting effects of HIV gp120 and proinflammatory cytokines, such as TNF-α. Furthermore, pre-treating GECs with curcumin was sufficient to block co-infection mediated *indirect* induction of the HIV promoter as well as TLR-mediated *direct* induction of HIV replication in T-cells.

In addition to the anti-inflammatory effects of curcumin, previous studies provide evidence that curcumin may block HIV by directly interfering with the HIV replication cycle. Curcumin-loaded apotransferrin nanoparticles have been successfully used to block the synthesis of HIV viral cDNA [44]. Other *in vitro* studies have demonstrated that curcumin can inhibit the enzymatic activity of recombinant, purified HIV-1 protease [45] and integrase [46], and can directly block the HIV-LTR promoter in T-cells [47–49]. Others have shown that combining curcumin with existing antiretrovirals, such as the protease inhibitor indinavir (IDV), reduced viral infectivity relative to IDV alone [50]. Collectively, these results suggest that curcumin may interact and block HIV replication via multiple, perhaps, redundant pathways and that there may be utility in supplementing current antiviral therapies with curcumin to reduce HIV-1 infection and replication.

Based on our results, we posit that protecting the mucosal barrier with curcumin may play a significant role in preventing mucosal transmission of HIV. Previously, we showed that gp120 interacts with TLR2, TLR4 and heparan sulphate moieties on primary GECs resulting in downstream induction of TNF-α, TJ disruption and barrier impairment [5]. This mucosal barrier disruption resulted in HIV translocation across the epithelium, presenting a mechanism by which HIV acquisition may take place [4]. In our current study, we found that curcumin pre-treatment prevented gp120-mediated disruption of the TJ proteins ZO-1 and occludin, and maintained TERs across endometrial and endocervical genital monolayers. Furthermore, pre-treating primary GECs with curcumin prevented gp120-mediated induction of barrier-breaking proinflammatory cytokines (TNF-α, IL-6) and HIV-target cell recruiting chemokines (IL-8, IP-10, RANTES). Together these results suggest that curcumin and other compounds, which reduce inflammation and maintain mucosal barrier integrity, may be useful candidates to test as prophylactic formulations to prevent HIV acquisition.

Curcumin may also play a significant role in preventing or reducing chronic immune activation. Microbial translocation due to mucosal barrier impairment is not only associated with HIV acquisition but is strongly believed to be one of the main contributors to chronic immune activation [51], characterized by increased inflammatory markers and immune cell activation that persists even in HAART-treated individuals [52, 53]. Immune activation is believed to be one of the main driving forces of CD4^+^ T-cell depletion and promoter of HIV replication [54]. Repeated exposure to HIV gp120 in mucosal tissues from shed virus or from unbound gp120 may continuously provide a source for generating inflammation in mucosal tissues that facilitates barrier breakdown and microbial translocation. Because immune activation is typically observed during the chronic stages of infection, the window of opportunity to intervene and limit these processes likely occurs during the early stages of HIV infection. Blocking HIV-mediated barrier disruption by curcumin may likely prevent microbial translocation and the initiation of immune activation, suggesting that curcumin treatment, may also contribute to decreasing the chronic inflammatory state that contributes to HIV pathology.

By suppressing replication in chronically infected T-cells, curcumin may provide a means of controlling HIV amplification during the early phases of infection. HIV crosses the mucosal epithelial barrier to establish a small founder population that then expands locally, possibly due to an influx of newly recruited target cells caused by upregulation of chemokines, prior to systemic dissemination [55]. These early events represent a window of maximum opportunity for interventions to prevent systemic infection. Our results show that in addition to protecting the mucosal barrier, curcumin may curb HIV by limiting local propagation and expansion in T-cells and by suppressing the local chemokine environment that may play a role in target cell chemotaxis. Our results also showed that repeated administration of curcumin worked best at suppressing HIV amplification in chronically infected T-cells, suggesting that therapies that continuously release curcumin may be novel tools by which HIV viral replication can be reduced in the FGT.

These results also support the notion that in addition to blocking HIV infection and replication, curcumin would be a useful compound to block bacterial and viral co-infections in the genital tract. Previous studies have characterized curcumin as a broad antimicrobial compound [21, 56]. The bactericidal capabilities of curcumin have been documented in the medical literature since the late 1980s [57] and have been anecdotally known for centuries. Previous studies have shown that curcumin inhibits NFκB pathways and production of IL-6, IL-8 and TNF-α following *N*. *gonorrhoeae* stimulation of the HeLa cervical epithelial cell line, and completely abolishes the adherence of bacteria to cells in late infection [58]. Furthermore, curcumin pretreatment significantly decreased HSV-1 infectivity and immediate early (IE) gene expression in HeLa cells [59] and provided significant protection in a mouse model of intravaginal HSV-2 challenge [60]. Using the Vero cell line, researchers also showed that curcumin and derivatives of the compound, containing gallium and copper metal, significantly suppressed HSV-1 replication *in vitro* [61]. Our results show that curcumin pre-treatment significantly impairs HSV-2 replication in primary human GECs, which is a more physiologically relevant model than transformed or immortalized epithelial cell lines, which were almost exclusively used in the past to examine the *in vitro* anti-herpetic activity of curcumin [59, 61]. Parallel experiments using inhibitors of inflammatory pathways known to be regulated by curcumin, showed that pre-treating cells with PDTC, an NFκB inhibitor, resulted in potent suppression of HSV-2 replication, suggesting that NFκB activation may play an essential role in HSV-2 replication in primary GECs, a notion that has been recently validated in epithelial cell lines [62]. Thus, our results suggest that curcumin could play a significant role in blocking co-infection mediated induction of HIV replication.

Our results add to the body of evidence indicating that curcumin could be a useful compound for decreasing HIV replication and perhaps even preventing HIV infection. However, clinical trials evaluating the efficacy of curcumin as a potential therapy for people living with HIV have had inconsistent results. In a clinical case study on three patients with HIV, two ART-naive and one who received conventional ART therapy, curcumin treatment resulted in a 10 to 10,000-fold reduction in blood viral load [63]. In this study, blood samples were tested for HIV viral load at baseline and following a regimen of 1 gram of curcumin, three times per day for 8 weeks. In contrast, a clinical trial examining the effectiveness of curcumin as an antiviral agent in 40 AIDS patients failed to find any therapeutic benefits. No evidence of curcumin-associated reduction in viral load was observed over the course of this short (8-week) trial, however, CD4 cells showed a slight increase in the high-dose curcumin group [64]. A possible reason for lack of effect in some of these studies could be due to curcumin’s poor bioavailability. Curcumin is poorly absorbed, rapidly metabolized/glucoronidated and systemically eliminated following oral administration [65]. Numerous efforts have been made to improve curcumin’s bioavailability by altering these features. The use of compounds that can block the metabolic pathway of curcumin is the most common strategy for increasing the bioavailability of curcumin, such as using piperine, a known inhibitor of hepatic and intestinal glucuronidation, which increased the bioavailability of curcumin in healthy human volunteers by 2,000% [66]. Other promising approaches to increase the bioavailability of curcumin in humans include the use of nanoparticles [67] or liposomes [68–70]. Other reasons why the previous clinical trial with curcumin failed to find any therapeutic benefits could be that the study was conducted on AIDS patients who may have already been past the point where curcumin would have been helpful. These studies did not examine whether curcumin had any effect on chronically infected individuals who had not reached the clinical stage of AIDS, or whether the compound had any role in decreasing chronic immune activation. Thus it remains to be seen whether the anti-inflammatory and replication suppressing roles of curcumin can work in tandem with conventional HAART therapy to provide better outcomes for HIV patients. Furthermore, no clinical studies have been conducted to examine prophylactic benefits of curcumin in preventing HIV infection. Our observation that curcumin protects the genital mucosal barrier against HIV would argue that prophylactic application of curcumin may be more effective as they could prevent HIV infection on mucosal surfaces.

In conclusion, our results indicate the promising potential of anti-inflammatory compounds such as curcumin, which protect the mucosal barrier and/or limit inflammation, unlike current antiretroviral therapies that target infection and replication. Inflammation may contribute to the acquisition or spread of HIV-1 infection, as well as contribute to the sequelea of chronic HIV infection. Thus, therapeutics designed to limit inflammation in the genital tract and/or systemically, may have a significant impact on HIV infection and disease progression.
